# Indicators for tracking programmes to strengthen health research capacity in lower- and middle-income countries: a qualitative synthesis

**DOI:** 10.1186/1478-4505-12-17

**Published:** 2014-04-12

**Authors:** Donald C Cole, Alan Boyd, Garry Aslanyan, Imelda Bates

**Affiliations:** 1 Dalla Lana School of Public Health, University of Toronto, Toronto, ON M5T 3M7, Canada; 2 Manchester Business School, University of Manchester, Manchester M13 9PL, UK; 3 TDR, World Health Organization, Geneva 27 CH-1211, Switzerland; 4 Department of International Public Health, Liverpool School of Tropical Medicine, Liverpool L3 5QA, UK

**Keywords:** Capacity building, Evaluation studies, Research personnel, Research support, World health

## Abstract

**Background:**

The monitoring and evaluation of health research capacity strengthening (health RCS) commonly involves documenting activities and outputs using indicators or metrics. We sought to catalogue the types of indicators being used to evaluate health RCS and to assess potential gaps in quality and coverage.

**Methods:**

We purposively selected twelve evaluations to maximize diversity in health RCS, funders, countries, and approaches to evaluation. We explored the quality of the indicators and extracted them into a matrix across individual, institutional, and national/regional/network levels, based on a matrix in the ESSENCE Planning, Monitoring and Evaluation framework. We synthesized across potential impact pathways (activities to outputs to outcomes) and iteratively checked our findings with key health RCS evaluation stakeholders.

**Results:**

Evaluations varied remarkably in the strengths of their evaluation designs. The validity of indicators and potential biases were documented in a minority of reports. Indicators were primarily of activities, outputs, or outcomes, with little on their inter-relationships. Individual level indicators tended to be more quantitative, comparable, and attentive to equity considerations. Institutional and national–international level indicators were extremely diverse. Although linkage of activities through outputs to outcomes within evaluations was limited, across the evaluations we were able to construct potential pathways of change and assemble corresponding indicators.

**Conclusions:**

Opportunities for improving health RCS evaluations include work on indicator measurement properties and development of indicators which better encompass relationships with knowledge users. Greater attention to evaluation design, prospective indicator measurement, and systematic linkage of indicators in keeping with theories of change could provide more robust evidence on outcomes of health RCS.

## Background

The need for all countries to generate and use health research in order to inform practice and policy decisions has become increasingly accepted over the last decade [1]. However, there remain gaps in the production of health research, particularly in many low- and middle-income countries (LMICs) [2]. Profiles to assess LMIC capacity for equity-oriented health research have been developed [3], resources assembled for health research capacity strengthening (RCS) [4], and ways forward proposed by leading African health researchers [5] and health systems organizations [6,7]. RCS has been defined as a “*process of individual and institutional development which leads to higher levels of skills and greater ability to perform useful research*” [8]. Experience has accumulated among those engaged in RCS for development in general [9], including case studies of health RCS [6], yet the heterogeneity and complexity of health RCS initiatives have hindered systematic assessments of effectiveness [10]. As one author has noted, “*We are at the early stages of knowing how best to identify, target and affect the many factors that are important for stronger research capacity. Furthermore, as RCS initiatives become more wide-ranging and complex, they become more difficult to monitor and evaluate.…. There is a clear need for improved strategies and the development of a tried and tested framework for RCS tracking*” [11].

Organisations that fund and manage research capacity strengthening initiatives, both nationally, e.g., the UK Collaborative on Development Sciences [9], and internationally, e.g., the ESSENCE on Health Research Initiative [12], have responded by collaborating to identify common issues relating to evaluating RCS programmes. Critical to making sense of RCS outcomes is the need to be explicit about the pathway by which change is to be brought about, i.e., the theory of change [13]. Indicators of the steps along the pathway from activities through outputs to outcomes can be linked within frameworks for evaluation of health RCS [14].

Currently, indicators or metrics are in widespread use in health programmes to monitor performance, measure achievement, and demonstrate accountability [15]. Generally accepted criteria for development evaluation involve use of Specific, Measurable, Attainable, Realistic and Timely (SMART) indicators [16]. Research impact evaluators have suggested including indicators not only of knowledge production and capacity development, but also of changes in health system policies, programs, and practices [17].

In the research reported here, we investigated reports of health RCS evaluations held by funders as a potentially rich source of untapped information. Our objective was to describe the design of health RCS evaluations, the nature of the indicators used, and the linkages among activities, outputs, and outcomes. We sought evidence to underpin the design of rigorous health RCS evaluations and the choice of indicators to be used for tracking progress and impacts, in ways which can transparently demonstrate value to all health RCS stakeholders – funders, research organizations, researchers, trainees, and research users.

## Methods

We adopted a qualitative approach to report identification, evaluation quality appraisal, indicator extraction, and synthesis. We consulted with stakeholders from LMIC health research funding agencies as part of a knowledge user oriented process [18]. Formal approval was obtained from the University of Toronto Health Sciences Research Ethics Board (#26837).

### Report identification

Our experience in reviewing proposals for health RCS, conducting and evaluating it, and searching the peer-reviewed literature led us to expect evaluations of a range of initiatives, from discreet projects, through portfolios of projects, to integrated long-term programmes. We initially consulted with funding agency members of the ESSENCE on Health Research initiative regarding report availability. Using a snowballing process, we invited other funders of LMIC research, known to support health RCS, to contribute health RCS evaluation reports. Eleven of 31 funding agencies contacted agreed to provide such reports, from which two authors agreed upon 54 reports of relevant health RCS evaluations that were publicly available, written in English, and produced since 2000. Each report was read by a pair of reviewers to assess the type of health RCS, funders, countries, detail available [19], and approach to evaluation. Sometimes more than one report was involved in evaluation of a health RCS initiative. Applying maximum variety sampling [20], we purposively selected 18 reports of 12 evaluations.

### Quality appraisal

Because of the growing emphasis on evidence of effectiveness, we appraised the quality of the evaluations. We derived the following quality appraisal questions from the Development Assistance Committee standards [16] and applied them to each evaluation report:

• Was the purpose of the evaluation clearly stated?

• Was the methodology described (including the analysis)?

• Were the indicators made explicit and justified?

In the methodology, we were particular interested in design, indicator measurement and collection, and bias. Our appraisal of the quality of indicators mentioned in the reports drew from SMART criteria (p10, Sn 2.9 of OECD standards) [16]. Two reviewers appraised each evaluation independently, providing brief justifications for their appraisals.

### Indicator extraction

We conducted a systematic framework analysis on the evaluation reports [21], extracting text relating to indicators used, and the context of that use. Many reports contained narrative descriptions of an activity, output, or outcome, which implied the nature of a corresponding indicator, while fewer explicitly defined indicators. Both descriptions and definitions were extracted and coded according to the categories in the ESSENCE Planning, Monitoring and Evaluation matrix [12] or to new categories that emerged. In order to promote learning and consistency in the extraction process, members of the research team each coded at least three reports, published by at least two funders, and relating to at least two evaluations, with each report being coded independently by two researchers. Discussion on coding of a smaller initial set fostered a common approach prior to coding all reports. Two authors independently extracted text from each evaluation report, checking consistency and resolving discrepancies through discussion, if necessary, by bringing in a third reviewer. We stopped when no new insights emerged from analysis of additional reports.

### Synthesis

We reviewed extracted material and created additional categories as needed. Given the importance of pathways consistent with theories of change, we attempted to identify and document links between aims and indicators and from activities through to outputs and outcomes. Clear descriptions of these links were unfortunately rare within any one evaluation. Therefore, we brought together examples of indicators and their contexts from several different evaluations, in order to illustrate the potential for such linkages. At several stages throughout the project our interim findings were discussed with the ESSENCE on Health Research initiative steering committee. Their feedback helped us to focus our analysis, and to validate and interpret our results [22].

## Results

The 12 evaluations were of health RCS initiatives covering the wide range we had expected. They were of different durations, conducted by different kinds of evaluators, at different stages of the initiative, and using a variety of evaluation approaches (Table 1) [23-40].

**Table 1:** Characteristics of international funders’ evaluations of LMIC health research capacity strengthening (RCS)

**Evaluations by international funder**	**Health RCS characteristics**		**Relation of evaluator to funder**	**Evaluation characteristics**	
	**Project, programme(s), organisation**	**Period (duration) covered by the evaluation**		**Timing**	**Main approaches/methods**
**ACU-CSC**[23,24]	RCS programme – with streams, health sector	1960+ (48 years)	Funder staff	Periodic review	Analysis of existing award data, alumni evaluation survey, 15 case studies, and 5 telephone interviews of selected scholarship recipients; impact assessment.
**Carnegie**[25]	RCS initiative with networks	2008–2010 (2 years)	Contract evaluation organisation	Mid-term	Desk review or initiative and network documents, interviews and focus groups with stakeholders (key staff and students within each network).
**Danida**[26]	Health research programmes of which health RCS is a part	1997–2006 (10 years)	Contracted evaluation organisation	Periodic review	Components were: a) country reports with visits; b) desk study review of projects; c) institutional questionnaires for Danish research groups; d) ‘internal’ [Danish organisations] individual staff questionnaires; e) ‘external’ [non-Danish other HIC funder] questionnaires and interviews; f) literature review of publications supported; g) evaluation document analysis; and h) health-related project database analysis.
**DfID**[27]	Project Health research council	2008–2010 (2 years)	External programme evaluation team	Mid-term review	Desk review of organisational, programme, and project documentation; site visit with interviews of stakeholders, beneficiaries, non‒beneficiaries, funders, and secretariat; in‒depth case studies of selected grantees and their institutions; and evaluation of the grants selection process.
**EDCTP**[28]	Health research partnership	2007–2009 (2 years)	Independent external panel	Periodic review	Documentation analysis, meetings/discussions and interviews with organisational representatives, questionnaire survey of researchers, site visit, conference attendance and country case study.
**IDRC**[29-32]	Health research programme with projects	Roughly 2001–2008 (7 years)	Contracted evaluation team	Special review	Conducted a gender audit at three levels – institutional, programmatic, and project (review of 15 projects) – through documentation review; search of guidelines and strategies of other organisations working on policy, health and gender issues; review of a previous internal gender survey; gender questionnaire to assess capacity development needs; and individual interviews with funder staff.
**NIH-FIC** (1) [33]	Health RCS programme	1992–2003 (11 years)	Contract evaluators	Periodic review	Outcome evaluation using NIH-FIC evaluation framework and FIRCA logic model. Administrative data collection and review, interviews with programme stakeholders, census surveys of the US principal investigators and international research collaborators, bibliometric analysis of publications, and site visits.
**NIH-FIC** (2) [34]	Health RCS programme	2002–2008 (6 years)	Contract evaluation team	Mid-term review	Programme implementation and preliminary outcomes. Data collection methods included two online surveys (GRIP awardees, unsuccessful applicants with scored applications). Supplementary data from administrative sources and databases, MEDLINE, and from interviews with US-based mentors, FIC staff members, and programme partners.
**NWO/WOTRO**[35,36]	Health RCS & health research programmes	2005–2008 (4 years)	(2008) Committee of three experts & two secretariat members (2009) Contract evaluators	Mid-term review	(2008) Background document review, discussions with programme coordinators, site visits with interviews, formulate recommendations, and discuss with Programme Committee. (2009) Not specified but included: programme document review, programme logic construction, projects’ progress reports analysis, and stakeholder interviews.
**Sida**[37]	Linked health RCS project funding (three routes)	1999–2005 (6 years)	Contract evaluators	Mid-term for re-formulation	Emailed questionnaires to institutions, individuals, and graduates. Interviews during site visits and evaluation seminar at main site.
**TDR –WHO**[38,39]	Organisation’s entire set of health RCS programmes	2000–2008 (9 years)	Contracted institute evaluation team	Periodic review	Questionnaires (individuals, research groups, and institutions), selected in-depth interviews, institutional site visits with stakeholder semi-structured interviews.
**Wellcome trust**[40]	Health RCS project – Consortium	2009–2011 (2 years)	Contract evaluation organisation	Mid-term (Second annual)	Real-time, monitoring and evaluation with mutually agreed framework of qualitative and quantitative indicators. Analysis in the light of all consortia within the programme of which this project is a part.

ACU-CSC, Association of Commonwealth Universities – Commonwealth Scholarship Commission (UK); Carnegie, Carnegie Corporation of New York through Science Initiative Group; Danida, Development cooperation activity, Ministry of Foreign Affairs (Denmark); DfID, Department for International Development (UK); EDCTP, European and Developing Countries Clinical Trials Partnership; ESSENCE, Enhancing Support for Strengthening the Effectiveness of National Capacity Efforts on Health Research Initiative; FIRCA, Fogarty International Research Collaboration Awards; GRIP, Global Research Initiative Program; HIC, High income country(ies); IDRC, International Development Research Centre (Canada); LMIC, Low- and middle-income countries; NIH-FIC, National Institutes of Health – Fogarty International Center (USA); NWO/WOTRO, Science for Global Development, Netherlands Organisation for Scientific Research; Sida, Swedish International Development Agency; TDR/WHO, Tropical Disease Research – World Health Organization.

### Quality of the health RCS evaluation designs

All evaluations had clear statements of their purpose or objectives, often with explicit terms of reference appended to the reports (see Quality Appraisal of Evaluations – Illustrative Examples below). Most evaluations used mixed method designs and drew on existing data or prior reports, often supplemented with site visits and/or interviews. The degree of complexity of the various evaluations reflected the complexity of the health RCS initiative; for example, the design of an evaluation concerning individuals in scholarship programs [23,24] was simpler than that used to evaluate changes in health economics capacity across an entire region [37]. Variability in evaluation design also related partly to the stage of the evaluation: a review early in the project cycle [27] was less complex than that of a long-running program undergoing a final stage review [38,39]. Several evaluators were constrained by the lack of a clear monitoring and evaluation framework [25], to help them orient their observations, and by the short time frame allowed for their review [26]. Though some reports were able to use historical comparisons [38,39], the majority were not able to draw on any baseline data [29-32], and only one evaluation considered (but did not use) a ‘control’ comparison [33]. These constraints limited assessment of change, its attribution to the health RCS programme, and potential estimates of effectiveness.

### Quality appraisal of evaluations – illustrative examples

#### Purpose of evaluation clearly stated

• To assist with the improvement of future development activities; to place tropical disease research in the existing landscape of health RCS [38,39].

• To appraise Swedish International Development Agency’s support to capacity building in the sub-Saharan Africa region. The most important purpose from the evaluators’ point of view was to provide stakeholders with the opportunity to learn about and develop the ongoing project [37].

• To assess implementation and preliminary outcomes, focusing on awardees careers; to guide a future outcome evaluation [34].

• To assess European and Developing Countries Clinical Trials Partnership (EDCTP) programme performance, including economic, social, and environmental impacts; address the role of EDCTP in the broader international research and development agenda; learn lessons and make recommendations for future initiatives [28].

#### Explicit evaluation design

• A feasibility study, including pilot tests, guided the evaluation survey design [33].

• Quantitative analyses of deliverables and a qualitative analysis of the process, perceived outcomes, and effects at regional, national, and institutional levels [38,39].

• Broad focus on all health-related alumni and impact of awards; in-depth focus on selected case studies and five alumni [23,24].

#### Data collection clearly described and validity checked

• Used qualitative interview recording, transcribing, and thematic coding. A self-assessment tool was used for research competency but its provenance was not explained [38].

• Interviews to solicit information on factors influencing post-grant careers; interviewees selected to balance gender, research interest, and nationality [38].

• Online surveys for awardees and unsuccessful applicants [34].

• Validity ensured by multiple data sources, triangulation, site visits, wide discussions to corroborate and validate information, and an iterative process throughout the evaluation [35,36,38-40].

#### Indicators explicit and justified

• Each bibliometric indicator provided insights into research quality, i.e., quantity of papers, citation rates, impact factor; norm-referencing [33].

• Indicators were stipulated in an evaluation framework and designed with stakeholders using intervention logic [40].

• Evaluation used EDCTP’s indicators, but limited by absence of any *a priori* formulated measurable indicators for the expected outcome set at the start of the programme [28].

#### Biases and limitations discussed

• The lack of a uniform monitoring and evaluation framework and reporting system resulted in collection of different types of data, and therefore different insights and conclusions [25].

• Limitations of using a self-assessment survey [25, p. 14] and the subjectivity of the evaluations and learning [40].

• Variables (e.g., linguistic, internet access) and potential biases in responses, recall, and classification were taken into account [33].

• The reasons for limited responses and the possibility of response bias were noted [34,37].

• Consideration was given to the feasibility of a comparative evaluation design and the need for longer-term and more rigorous design to assess outcomes and impact of Global Research Initiative Program [34].

• Unavailability of original documentation [23,24,29-32].

• Lack of pre-determined measurable indicators and independently verifiable data necessitated an opinion-based retrospective evaluation [28].

The evaluations surveyed individual grantees, institutional representatives, or relevant key informants at national and international levels. Questionnaires were all crafted specifically for the evaluation, i.e., no existing instruments with known properties or prior validation were used, yet only one evaluation report describing a formal pilot to test the questionnaire was used [33]. Half of the evaluations explicitly addressed potential biases or other threats to the integrity of the evaluation, with some noting low response rates. Some cited the importance of site visits and other means to triangulate reports from grantee respondents, and a few described iteratively re-visiting different groups to obtain feedback on their emerging findings.

### Indicators used for tracking progress in health RCS initiatives

The extent of indicator description and depth of justification for the choice of indicators varied widely between evaluation reports. Indicators were often linked to specific objectives, attainable and realistic for the programme, and many were timely for programme monitoring (SMART criteria). Developing indicators that were ‘measurable’ seemed to be more of a challenge. Some reports referred to “*measuring progress using testable goals – relevance, governance, efficiency and effectiveness*” [23], but did not provide an explicit definition of these terms. A few evaluations did include indicators which involved considerable measurement work, e.g., on bibliometric indicators of the quality of research performed by grantees [33]. Other evaluations explicitly linked indicators to intervention logic frameworks (see Quality Appraisal of Evaluations – Illustrative Examples above). Although no single evaluation provided enough information to enable us to describe an explicit pathway of activities to outputs and outcomes, it was possible to link common indicators across evaluations. Specific examples follow to illustrate the context in which the indicators were used at different levels.

#### Individual level indicators

Indicators relating to training in research skills for researchers and also other personnel, such as data managers and laboratory staff, were common (see Table 2). Indicators of training in areas relevant to professional skills (e.g., research management) and of training quality (e.g., PhD education) meets international standards [40] and researcher/student satisfaction, were noted in some reports [34-36].

**Table 2:** Potential pathways to change: indicators of outputs and outcomes linked to activities primarily at the individual level

**Research skills training activities:** PhDs, MScs, scholarships, fellowships, and salary supplementation. Training of research support staff, i.e., data managers, research laboratory personnel, statisticians, and research managers.	
**Outputs**	**Outcomes**
Feedback from recipients about career prospects.	Development of research skills, i.e., identification of a research problem, analytical review of a scientific article, research proposal, and scientific report writing.
Quality of training.	
Balance between training in research methods (i.e., protocol, methods, collection and analysis), research process (i.e., writing, communication, knowledge transfer), and advocacy, promotion, negotiation, and resource mobilisation.	Quantitative and qualitative evidence of the effectiveness of the awards (from survey about careers, achievements, and impact).
	Evidence that awardees returned to active and independent research in LMICs.
	Reasons why trainees did not return/stay in LMICs (e.g., poor career prospects; no opportunity to use skills).
	Development of sustainable research collaborations.
	For HIC researchers, improved understanding of international research issues and increased desire to collaborate with researchers in developing countries.
	New research funding obtained.
**Mentoring activities:** Individual support for developing skills in research and supervision.	
**Outputs**	**Outcomes**
Number of trainees with a mentor.	Number of grantees working as senior researchers and their location (e.g., academia, in government agencies, or private sector).
Knowledge of reasons for lack of career development, i.e., lack of resources, supervision, and collaborators.	
	Percent of time spent on research activities.
**Scientific conference and workshop activities:** Health Economics Conference, EDCTP Forum, networking, sharing with colleagues, and policy makers.	
**Outputs**	**Outcomes**
Number of meetings/workshops attended pre- and post-funding.	Research by awardees published in conference proceedings.
	Invitations to speak at meetings. Honours, awards, esteem, expanded social networks.
	Membership and/or leadership role (e.g., president, chair, secretary, editor) in professional societies, advisory groups or scientific journal.
**Course and curricula development activities:** Short courses/diplomas/degrees in research skills and methods, and scientific topics developed in response to a needs assessment and embedded within the university.	
**Outputs**	**Outcomes**
Partnerships used for course design, student supervision, mentoring, and bilateral recognition of credits.	Secondary benefits to students through training, travel and education opportunities made them ‘diffusers’ of new techniques between institutions.
Courses (e.g., masters, PhD) run by university consortia promoted relationships between universities and/or across specialities (e.g., health economics).	
Database of courses; attendance register.	

EDCTP, European and Developing Countries Clinical Trials Partnership; HIC, High income country; LMIC, Low- and middle-income countries.

Some reports included indicators that assessed equality of award allocation by utilising data which had been disaggregated by gender, nationality, country income level, discipline, and level of award [34,38,40]. One evaluation specifically focused on how gender-related indicators could become more part of research funding, training, and reporting [29]. Other equity-related disaggregations, e.g., by socio-economic status within the country or potentially excluded groups, such as ethnic minorities or aboriginal peoples [16], were only apparent in one evaluation [28, p. 85]. From a North–South equity perspective, one evaluation noted “*Most project coordinators and project leaders or principal investigators are African researchers (55.5*%*), with good representation of female researchers: 40*% *in AIDS projects and 25*% *in TB and Malaria projects*” but the benchmark for such judgements was not clear [28].

Some evaluations included indicators for trainee mentoring, noting low mentor to trainee ratios, with intense competition for senior supervisors among the many research projects funded by international donors [34]. Job outcome indicators were pertinent to several health RCS activities and were very much affected by context, e.g., the lack of career opportunities/structures for post-doctoral students resulting in a high proportion of PhD graduates not continuing active research careers [35,36].

#### Institutional level indicators

Several evaluations linked support for individual grantees to institutional research strengthening [35,36,40]. Others retrospectively analysed funding allocations according to location and characteristics of the recipient institutions [34]. Indicators used in an evaluation of a PhD and MSc Fellowship programme identified a lack of institutional guidance on criteria for student selection and a lack of linkage between training support and engagement in research [35,36]. Other indicators focused on the institutional capacity to mentor more junior researchers (as distinct from supervision of research students), to help returning graduates start research (e.g., an improved information and communication technology system to facilitate communications with colleagues globally), and to support active investigators (Table 3).

**Table 3:** Potential pathways to change: indicators of outputs and outcomes linked to activities primarily at the institutional level

**Human resources strengthening activities:** Staff training and recruitment (e.g., data management, laboratory scientists), including salaries. Strengthening inter-staff and inter-student relationships. Promoting inter-disciplinarity, diversity, and specialization.	
**Outputs**	**Outcomes**
Numbers of potential supervisors.	Recruitment and retention of researchers, supervisors, and core staff.
Capacity to mentor junior researchers, take on	
leadership and inspirational roles.	Clear research career paths/possibilities.
Institutional destination/return home of researchers and graduates.	Involvement of research managers in the collaboration/network.
**Activities for strengthening research infrastructure and management:** Support for infrastructure (e.g., laboratory facilities, equipment, and maintenance; libraries, IT, computers). Setting up ethical review boards, engagement of stakeholders and secretariats. Improved governance, planning, strengthening of financial reporting, institutional evaluation capacity, and gender analysis.	
**Outputs**	**Outcomes**
Establishment of cross-cutting projects, sharing of equipment (e.g., fridge, freezer, thermocycler, microscopes, centrifuge, and computer), staff (e.g., lab technicians), and systems (e.g., data management) facilitates integration of research activities.	Better access to resources (e.g., staff, libraries, journals, equipment).
	Research staff satisfied with institution’s research services (i.e., workplace, library, internet access, journal access, lab facilities, purchasing system, maintenance, human resources).
Standard operating procedures, quality assurance mechanisms.	
	Improved management and administrative capacity and technical capacity (e.g., for lab quality control, trial monitoring services, data management, and data analysis support).
A research support centre, scientific steering committee, institutional governance structure, and organisational chart.	
Commitment to or implementation of strategic	
planning, management, new policies, resource allocations.	Achievement of international accreditation, e.g., of laboratories able to attract private funding as well.
Evidence of a transferable partly self-sustaining model (salaries externally supported) for Research Support Centre.	
**Scientific collaboration activities:** Promotion of collaborations for North–South and South-South and/or regional partnerships, sometimes restricted to existing grantees, or projects led from the South.	
**Outputs**	**Outcomes**
Formal agreements, including for data sharing.	Collaborations characterised by trust and commitment, and continue after award concludes.
Site inspections, meetings together.	
Joint PhD students, projects, and technologies shared between collaborators.	
	Benefits for northern institutions (i.e., understand LMICs health system, engage with research and training institutions).

Indicators related to research infrastructure and management activities focused on ‘hard’ infrastructure (e.g., libraries, lab equipment) and ‘softer’ systems (e.g., implementation of new routines, policies, resource allocations and systems of compensation [37], leadership of funding proposals to attract research funding and implement and manage research [35,36], and the development of organisational learning mechanisms) [33]. A few evaluations highlighted missed opportunities such as the limited sharing of donated equipment, materials, and techniques [35,36]. Indicators of institutional collaborations used in some evaluations included local ownership [26], regional partnerships [26,27], and enhanced visibility of the institution in the national and international research communities [34].

#### National/international level indicators

Indicators of activities with national policy makers, regional organizations, or networks captured components of the system in which individual and institutional health RCS were embedded (Table 4) [6,14]. Stakeholder engagement and research uptake indicators included systematic identification of potential users of research for early engagement [34,40], a comprehensive communication strategy [40], and appropriate tailor-made tools for dissemination of research [33,38]. Indicators of the capacities of research users and policy makers to utilise research information were rare but included skills in acquiring research information, assessing its quality, and using it for decision-making [37]. Involvement of non-scientific communities was used as an indicator of embedding research partnerships within public health structures [37]. Important indicators of national research capacity were the commitment of Ministries of Health to research and the development of national research councils with explicit national research priority-setting processes and legal frameworks for research [27,34].

**Table 4:** Potential pathways to change: indicators of outputs and outcomes linked to activities primarily at the national-international level

**Engagement and communication activities for research uptake:** Engagement with private and non-health organisations, NGOs, HIV programmes, research institutions, health ministries, regulatory authorities. Using journals, press, magazines, conferences/workshops, networks, face-to-face interaction, websites, consensus reports, policy briefs, newsletters.	
**Outputs**	**Outcomes**
Skills development program from public-private-academic partnerships.	Advocacy resulted in enhanced health RCS effort, or enhanced knowledge about neglected topic diseases (e.g., fish-borne zoonotic parasites).
Systematic plan for acquiring and using research information, and for sharing and transferring knowledge.	
	Knowledge about focus of health RCS efforts – tend to be more on researchers and less on research users.
Media articles (i.e., press, magazines, reports, website).	Partnerships for research dialogue (e.g., with policymakers, research users, decision makers national authorities, professional groups, private sector, NGOs, civil society) at local, regional, and international levels.
Communication/knowledge management strategy	
Trends in website hits.	
**Activities to develop national health research systems or scientific councils:** Promote financial sustainability in regional research activities.	
**Outputs**	**Outcomes**
Map of national research system.	Strong commitment and active engagement by national health research institutions and health ministries to review progress and determine research priorities.
	Knowledge about contribution (or not) of national agencies to development of effective national health research system and in creating demand for research.
	External funds provided more accessibility and flexibility than local funds.
**Networking activities for researchers and/or research users:** Facilitation of collaborations and large-scale networks, sometimes through multi-disciplinary workshops, curricula, meetings, and seminars.	
**Outputs**	**Outcomes**
New programme and partnership for research to strengthen links between universities and policy making (e.g., systematic reviews for research).	Impact on policy, practice, and knowledge at different levels (i.e., international, regional, national, district level) and on health and non-health sectors, through research and policy networks.
Project staff contributed to evaluations of health centres and systems and to motivating medical staff.	Estimated impact on disease control and prevention.
	Harmonised regional research activities.
North–South and South–South networking activities.	
Active committees with institutional representation in each member country.	
Commitment and communication with the Northern and among Southern partners.	

Development of trans-disciplinary platforms and networks of researchers or institutions were key indicators of the ability to assemble a critical mass of researchers [26,27,40]. The promotion of the financial sustainability of research capacity within a country or region, sometimes through involvement of private partners, was another indicator [34]. Network leaders were identified as graduates from previously funded programmes, a long term indicator of one programme’s impact [34]. An indicator of stability of co-operation across partner institutions was lack of dependence on specific individuals in a context of high personnel turnover [26,34]. Whether a network was dysfunctional or smoothly run, whether feelings of injustice and insecurity were developing [26], and the existence of rules around ‘competition-collaboration’ [34] were all indicators of the quality of network functioning. One evaluation of a programme which funded multiple networks noted that most networks did not have information ‘at their fingertips’ and some could not obtain output level data. It urged the use of a more formal monitoring and evaluation framework grounded in each individual programme’s theory-in-use and program logic [26].

## Discussion

### Indicator coverage

Our systematic analysis of diverse international health research funders’ evaluations uncovered a broad set of indicators including metrics available to measure return on investment in health research [17]. Many of the evaluations used a subset of indicators among those identified by the ESSENCE on health research initiative including curricula developed, courses run, researchers trained, scientific collaborations initiated, and partnerships strengthened. Given the global focus on health equity [41], the rarity of disaggregation of indicator data according to equity categories was concerning. The Ford Foundation’s work on active recruitment of those from disadvantaged backgrounds [42], and NIH-FIC’s Career Track’s inclusion of ‘minority type’ (Celia Wolfman, personal communication) hold promise.

Missing in the evaluations were some important constructs relevant to health RCS, particularly ongoing relationships among RCS stakeholders to facilitate conduct and use of research [43]. Further, nomenclature was highly variable for the national/international level – terms included societal, macro, environment, and network, perhaps reflecting the systems nature of much health RCS [44]. Use of the term ‘local’ to describe that which is not global is not particularly helpful in thinking about the scale of health RCS efforts, as it can refer to scales from a community, through municipalities, districts, provinces or regions within a country, and nations to multi-country regions, e.g., East Africa. Greater attention should be paid to clarifying scale, perhaps separating out three components – provincial-national research environment, international-global research environment, and research networks – in order to facilitate greater clarity of relationships between indicators and consistency in cross case comparisons [6,7].

### Indicator quality

Comments on quality of indicators were rare, despite Development Assistance Committee standards, and only a few individual indicators met most of the SMART criteria [16]. The quality challenges may reflect the division of responsibility for collecting indicator-related data among funders, institutions and researchers implementing health RCS, and evaluators. They may also reflect the limited investment of time and resources in evaluations, relegating them to more of a milestone monitoring role than a key ingredient for determination of equity, effectiveness, or efficiency. Each stakeholder may be interested in different indicators on account of their differing roles in assessing research impact [45]. Stakeholders should therefore be involved in early planning regarding the selection and quality of indicators to be used [46].

### Health RCS contribution assessment

Virtually all evaluations were retrospective in nature, with only a few [33,40] engaging in the kind of forward planning to promote applicability of indicator selection over time and rigour of evaluation designs [46]. Few evaluations systematically considered assumptions, pre-conditions, or measurement challenges, confounders or co-interventions, all of which are needed to clarify causality. Explicit use of theories of change [13] with delineation of pathways linking indicators within explicit frameworks [14] was rare, perhaps because of the limited attention to mechanisms by which health RCS initiatives might effectively address problems identified and bring about the hoped-for changes [46]. Such gaps undercut assessment of the contribution of health RCS programmes to longer term impacts [47].

### Limitations of our study

Not all health RCS funders whom we approached provided reports. Further, we could not undertake detailed analysis on a large number of evaluations due to the labour intensive nature of data extraction and analysis. Nevertheless, the evaluations we did analyse covered a broad range of countries, types of health RCS initiatives, international funders, and contexts. Many common themes emerged during our analysis, particularly later in our analysis process, as we reached saturation, suggesting that incorporating additional evaluations would not yield substantially new information. Allocation of extracts from evaluation narratives and indicators to the various framework analysis categories was occasionally only resolved through discussion. Most evaluations captured only one point in the life cycle of a health RCS initiative – only two tracked health RCS longitudinally [23,24,38]. Similarly, only a few encompassed the contributions of a range of health development efforts, research programs, and RCS initiatives, to the gradual emergence of a health research system, as has been possible in case studies taking a longer term view [48].

### Directions for evaluation of health RCS

The strengths and weaknesses of the health RCS evaluations which we analyzed likely reflect those in the broader field of evaluation of research for development. Certain development funders are committed to “*strengthening the evidence base for what works or does not work in international development as well as developing and strengthening evaluation research capacity within the UK and internationally*” [49]. Where health RCS is integrated within a research program, an adequate proportion of the program budget should be allocated to quality evaluation, e.g., US federal guidelines suggest 3% to 5% for evaluation activities [50]. Rigorous evaluation design could draw on development evaluation efforts by organizations such as the International Initiative for Impact Evaluation (http://www.3ieimpact.org) and the Network of Networks on Impact Evaluation (http://nonie2011.org/). Building on the mixed methods work synthesized here, systematic attention to indicator framing, selection, measurement, and analysis, could occur while maintaining flexibility and revisiting indicators as health RCS proceeds [46,51]. We have formulated these potential directions as a set of recommendations for which different stakeholders in health RCS could show leadership (Table 5).

**Table 5:** Recommendations for different stakeholders to improve health RCS evaluation*

**Recommendation**	**Funding agencies**		**Priority decision-makers**		**Producers**		**Users**		**Evaluators**
	**International**	**National**	**International organizations**	**National research councils**	**Institutions (universities, research institutes, NGOs), networks**	**Researchers (established and learning)**	**International organizations**	**National and sub-national health services**	
Adequate allocation of resources to quality evaluation research alongside investments in the quality of the science, scientists, and science communication.	+++	++		++					
Systematic attention to indicator framing, selection, measurement (multiple data sources and valid standards to enhance quality), and analysis.	+	+			++	++	+	+	+++
Development of indicators which better encompass relationships with knowledge users.			++	++	++	++	++	++	+++
Disaggregation of indicator data according to equity categories.	+	+			++	++	++	++	+++
Systematic consideration of assumptions, pre-conditions, or measurement confounders associated with the evaluations.						++			+++
Greater attention to evaluation design, use of clear conceptual frameworks, systematic linkage of indicators in keeping with theories of change.	+	+				++			+++
Development of comprehensive, prospective systems for health RCS indicator monitoring and evaluation, in which long-term impact is considered throughout the entire project cycle.	++	++	+	+	++	++	+	+	++
Separation out of three components of the upper level– provincial-national research environment, international-global research environment, and research networks.	++	++	++	++	+	+	+		+++

*Role designated as + small, ++ medium, or +++ large.

## Conclusions

Our research has synthesized new knowledge about evaluation designs and associated indicators that can be tracked in different contexts for different health RCS initiatives, tailored to the particular aims of an initiative. The use of more rigorous designs and better measurement within clearer evaluation frameworks should produce the kinds of robust evidence on effectiveness and impacts that are needed to better justify investments in health RCS.

## Abbreviations

EDCTP: European and Developing Countries Clinical Trials Partnership; LMICs: Low- and middle-income countries; RCS: Research capacity strengthening; SMART: Specific, Measurable, Attainable, Realistic and Timely.

## Competing interests

The authors declare that they have no competing interests.

## Authors’ contributions

Each author participated in the design, data identification and collection, analysis and interpretation, writing, and revisions. All authors read and approved the final manuscript.
